# Effect of Dietary Benzoic Acid and Oregano Essential Oil as a Substitute for an Anti-Coccidial Agent on Growth Performance and Physiological and Immunological Responses in Broiler Chickens Challenged with *Eimeria* Species

**DOI:** 10.3390/ani14203008

**Published:** 2024-10-17

**Authors:** Joycy Seiba Khukhodziinai, Pradip Kumar Das, Joydip Mukherjee, Dipak Banerjee, Prabal Ranjan Ghosh, Anil Kumar Das, Indranil Samanta, Ruma Jas, Samiran Mondal, Amlan Kumar Patra

**Affiliations:** 1 Department of Veterinary Physiology, Faculty of Veterinary and Animal Sciences, West Bengal University of Animal and Fishery Sciences, Kolkata 700037, West Bengal, India; jseiba07@gmail.com (J.S.K.); joyphy@gmail.com (J.M.); dipakndri@gmail.com (D.B.); probal.gh@gmail.com (P.R.G.); anilnrcp1@gmail.com (A.K.D.); 2 Department of Veterinary Microbiology, Faculty of Veterinary and Animal Sciences, West Bengal University of Animal and Fishery Sciences, Kolkata 700037, West Bengal, India; isamanta76@gmail.com; 3 Department of Veterinary Parasitology, Faculty of Veterinary and Animal Sciences, West Bengal University of Animal and Fishery Sciences, Kolkata 700037, West Bengal, India; 4 Department of Veterinary Pathology, Faculty of Veterinary and Animal Sciences, West Bengal University of Animal and Fishery Sciences, Kolkata 700037, West Bengal, India; 5 Department of Animal Nutrition, Faculty of Veterinary and Animal Sciences, West Bengal University of Animal and Fishery Sciences, Kolkata 700037, West Bengal, India; 6 American Institute for Goat Research, Langston University, Langston, OK 73050, USA

**Keywords:** coccidia infection, feed additive, hematobiochemical profile, immunity, intestinal bacteria, phytocompound, poultry

## Abstract

To overcome the antimicrobial residues in food, benzoic acid (BA) and oregano essential oil (OEO) are used in the broiler chicken industry. Independently, both exerted anticoccidial and antimicrobial actions and improved growth performance in broiler chickens. Their effect may be multiplied when they are used in combination. This present study was carried out to evaluate the efficacy of dietary BA and OEO alone or in combination as a substitute for a commercial coccidiostatic drug on growth performance and physiological and immunological responses in broiler chickens challenged with *Eimeria* species. A total of 252 unsexed 1-day-old broiler chicks were equally allotted to 36 pens, each pen containing seven chicks. The pens were randomly assigned to six treatments with six pens (replicates) for each treatment (*n* = 6)—(i) negative control, (ii) positive control, *coccidia*-challenged and non-treated, (iii) supplemented with salinomycin (an anti-coccidial drug) at 60 mg/kg of feed and *coccidia*-challenged, (iv) supplemented with BA at 500 mg/kg of feed and *coccidia*-challenged, (v) supplemented with OEOat 500 mg/kg of feed and *coccidia*-challenged (OEO), and (vi) supplemented with BA at 500 mg/kg of feed and OEO at 500 mg/kg of feed and *coccidia*-challenged (B&O). The liver enzymes and thyroxine and creatinine levels were not affected (*p* > 0.05) both in coccidia-challenged and supplemented chickens. The BA and OEO applied separately or in combination (B&O) significantly (*p* < 0.05) reduced gut pathogenic bacteria (*Salmonella* and *Escherichia coli*) and *Eimeria* spp., and concurrently enhanced (*p* > 0.05) the *Lactobacillus* population with better body weight gain, improved feed utilization, and superior hematological values. It also up-regulated (*p* > 0.05) the interferon-γ gene expression and down-regulated (*p* < 0.05) the interleukin-10 and Toll-like receptor-4 gene expression to protect the chickens from inflammatory reactions, which were not demonstrated in salinomycin-treated birds. The B&O supplementation increased (*p* < 0.05) the immune system by enhancing *Eimeria*-specific immunoglobulin Y titer and lymphocyte proliferation response. This study suggests that the combined application of OEO and BA can substitute for a commercial anti-coccidial agent (salinomycin) in controlling coccidiosis as well as improving growth performance, gut health, and immune responses in broiler chickens with a means of antimicrobial-resistant free food products.

## Simple Summary

This present study evaluated the efficacy of dietary benzoic acid (BA) and oregano essential oil (OEO) separately or together as a substitute for a commercial coccidiostatic drug (salinomycin) on growth performance and physiological and immunological responses in broiler chickens challenged with *Eimeria* species. It was found that the BA and OEO applied alone or in combination significantly reduced gut pathogenic bacteria (*Salmonella* and *Escherichia coli*) and *Eimeria* spp. and concurrently enhanced the *Lactobacillus* population with better body weight gain, improved feed utilization, and superior hematological values. It boosted the immune system by enhancing *Eimeria*-specific immunoglobulin Y titer and up- and down-regulated various immune gene expressions to protect the chickens from inflammatory reactions that were not demonstrated in salinomycin-treated birds. This study suggests that the combined application of OEO and BA can substitute for salinomycin in controlling coccidiosis as well as improving growth performance, gut health, and immune responses in broiler chickens.

## 1. Introduction

Avian coccidiosis, caused by *Eimeria* spp., is one of the vulnerable enteric diseases and is endemic in vast areas of the tropical and subtropical regions [1]. It reduces growth performance affecting chicken meat production and accounts for about 30% of the total expenses on medications and other pharmacological products used to control poultry diseases [2]. Out of seven recognized species, *E. acervulina*, *E. necatrix*, and *E. tenella* cause serious disease outbreaks, having no cross-immunity among them [1]. These intracellular protozoa damage gut tissues and facilitate the proliferation of intestinal pathogens, leading to reduced body weight (BW) gain due to decreased feed intake [3]. Merogony, gametogony, and sporogony occur during the life cycles of *Eimeria* species within the host and environment, respectively [4]. After shedding, the oocysts remain viable; hence, outbreaks of coccidiosis are primarily linked to intensive rearing conditions and environmental factors [5]. To control avian coccidiosis, many commercial anticoccidials, especially salinomycin, have been utilized extensively against various *Eimeria* species leading to the emergence of parasites resistant to anticoccidials, a great concern to environmental issues in this present scenario [6,7]. Consequently, commercial antimicrobial-free poultry feed supplements have been promoted for the safe animal food production process as an alternative strategy for managing coccidiosis by enhancing the chickens’ innate immune systems [8,9].

Dietary acidifier benzoic acid (BA) is one such compound that improves gut integrity [10] and modulates gastrointestinal function by affecting pH-sensitive gut pathogens [11]. The antioxidative nature of BA and its ability to scavenge reactive oxygen species (ROS) [12] make it a natural antimicrobial agent that can help mitigate coccidiosis [13], along with an immunomodulation effect [14]. BA has been shown to improve average daily weight gain and feed conversion ratio (FCR) due to better nutrient digestibility in *Eimeria*-infected birds [15]. Oregano essential oil (OEO), a hepatoprotective phytochemical, has been used as an environmentally friendly alternative to antibiotic growth promoters to improve intestinal health, nutrient utilization, and growth performance in broiler chickens [16], including under *Eimeria*-infected conditions [17,18], inducing innate immunity and improving FCR [19]. It was also reported that OEO improves the hemato–biochemical profile [20], induces phagocytic activity [21], effectively reduces *Eimeria* population in the gut of chickens [17], and elicits a better immune response than salinomycin [22].

It was postulated that the dietary use of both BA and OEO together would effectively exert anticoccidial and antimicrobial actions and promote growth performance while preserving physiological homeostatic mechanisms in broiler chickens infected with *Eimeria* and addressing the antimicrobial-resistant animal food production process issues. However, a research gap exists for the appraisal of BA and OEO on growth performance, coccidia loads, immunity, and gut health compared with commercial anticoccidials in different farm conditions. We hypothesized that BA and OEO could serve as alternatives to synthetic anticoccidial chemicals without compromising production performances in coccidia-challenged conditions. Consequently, the current experiment was conducted in the environment of a small-scale poultry farm to evaluate the efficacy of BA and OEO, either alone or together, on growth performances, hemato–biochemical profile, and immune responses in *Eimeria*-infected broiler chickens to combat the environmental challenges concerning antimicrobial residue in the food chains.

## 2. Material and Methods

### 2.1. Experimental Animals and Design

The experimental design and protocol were approved by the Institutional Biosafety Committee (No: FVAS/Micro-IBSC/04/22-23) and Institutional Animal Ethics Committee of West Bengal University of Animal & Fishery Sciences, Kolkata, India (No. 763/GO/Re-S/ReRc-L/03/CCSEA/66/2023-24).

A total of 252 unsexed Cobb-500^TM^ (Cobb-Vantress, Inc., Siloam Springs, AR, USA; www.cobb-vantress.com; accessed on 26 January 2022) 1-day-old broiler chicks with initial BW of 42.0 ± 2.17 g were randomly and equally divided into 36 pens, each pen containing seven chicks (Table 1). The pens were randomly assigned to 6 treatments with six pens (replicates) for each treatment (*n* = 6), viz., (i) negative control (NC); (ii) positive control, *coccidia*-challenged and non-treated (PC); (iii) supplemented with a commercial anti-coccidial drug, salinomycin (Coxistac^®^, Phibro Animal Health Corporation^TM^, Teaneck, NJ, USA; marketed by Zenex Animal Health India Pvt. Ltd., Ahmedabad, India) at 500 g/kg to obtain an effective dose of 60 mg salinomycin per kilogram in the finished feed [23] and *coccidia*-challenged (Sal); (iv) supplemented with BA (Hi-LR^TM^, Himedia Laboratories Pvt. Ltd., Thane, India) at 500 mg/kg of feed (BA) following the recommendation of EFSA FEEDAP Panel [24] and *coccidia*-challenged; (v) supplemented with OEO extracted from *Origanum compactum* (Allin^TM^ Exporters, Noida, India) at 500 mg/kg of feed [22] and *coccidia*-challenged (OEO); and (vi) supplemented with BA at 500 mg/kg of feed and OEO at 500 mg/kg of feed and *coccidia*-challenged (B&O).

Birds in all groups, except the NC, were challenged with the live sporulated oocysts (Livacox^®^Q; Biopharm, Research Institute of Biopharmacy and Veterinary Drugs, Pohoří, Czech Republic; marketed by Hester Bioscience Ltd., Ahmadabad, India). Each bird received an oral dose of 0.1 mL, 10 times the recommended vaccine dose, consisting of 3000–5000 live sporulated oocysts in each attenuated line of *E. acervulina*, *E. maxima*, and *E. tenella*, and 1000 live sporulated oocysts of the attenuated line of *E*. *necatrix* in a 1% *w*/*v* aqueous solution of chloramine B. The dose of Livacox^®^Q was increased tenfold from its usual vaccine schedule to induce mild infection, following protocols established by Nawarathne et al. [10] and Lu et al. [25].

Birds in all groups were provided with the basal concentrates diet (Table 2) and received group-wise supplementation from day 1 with the gradual replacement of starter (1 to 14 days), grower (15 to 28 days), and finisher (29 to 35 days) diets under standard operating procedure to meet or exceed the minimum nutrient requirements recommended by the Cobb 500 Broiler Performance and Nutrition Supplement [26]. The supplemented ingredients were thoroughly mixed with the mash feed on a weekly basis using a feed mixture, and diets were kept in air-tight polythene bags.

Similar standard management practice was followed for all the groups rearing at the experimental farm under the West Bengal University of Animal and Fishery Sciences, Kolkata, India (at 22°34′ N and 88°24′ E). The farm was disinfected with potassium permanganate solution (1:1000) 15 days before the arrival of chicks and fumigated with potassium permanganate and formaldehyde solution. The feeding and watering utensils were thoroughly cleaned and sanitized. The chicks were given continuous light for the first two days of brooding, and they quickly adapted to their new schedule of 23 h of light and 1 h of darkness. Environmental temperature and relative humidity were measured using dry-bulb and wet-bulb thermometers (Omsons Glassware Germany, Haryana, India) throughout the study period. The temperature humidity index (THI) was calculated according to the method described by Tao and Xin [27]. The mean THI was 26.75, which falls within the comfort range for broilers [28]. All birds received vaccinations against infectious bursal disease at 12 days of age and Newcastle disease at 5 and 20 days of age.

### 2.2. Variables Studied

#### 2.2.1. Growth and Feed Intake

Body weights were measured pen-wise with a digital weighing balance on days 7, 14, 21, 28, and 35 (at 6 a.m.). Weekly BW gain was calculated by subtracting the initial BW from the final BW for a particular week. The average daily BW gain (ADG) was calculated by dividing the BW by the age reared in days. The average feed intake was calculated by subtracting the cumulative unconsumed daily feed from the offered feed every week. The average daily feed intake (ADFI) was calculated by dividing the feed consumed by the number of days, and feed conversion ratio (FCR) was obtained by dividing feed intake by the live weight gain during the period.

#### 2.2.2. Sampling

Hemato–biochemical, including enzymes and hormones; fecal microbes and oocysts; antibody titer; and immunological variables were studied at weekly intervals after coccidia challenge up to day 35, i.e., on day 21 (7 days post-infection, DPI, week 3), day 28 (14 DPI, week 4) and day 35 (21 DPI, week 5).

Blood samples (4 mL) were collected from wing vein (*n* = 4 per replicate) on 7, 14, and 21 DPI. The samples were distributed equally into (i) ethylenediamine tetra-acetic acid (EDTA) coated vacutainer vials (Hi-media Laboratories Pvt. Ltd., Thane, India) for hematological and immunological studies, with the plasma separated from the remaining blood and stored at −20 °C for further analysis, as well as (ii) non-EDTA-coated vacutainer vials for serum collection for the antibody titer study.

Birds were slaughtered (*n* = 2 per replicate) by cervical disarticulation to collect cecal contents for oocyst counting on 7, 14, and 21 DPI as well as jejunum tissue samples for the immune regulatory gene expression study on 7 and 21 DPI.

#### 2.2.3. Hemato–Biochemical, Enzyme, and Endocrine Profiles

Hemato–biochemical, enzyme, and endocrine profiles were measured on 7, 14, and 21 DPI from four birds in each replicate (*n* = 4 per replicate) across six experimental groups. Hemoglobin (Hb) percentage following the cyanmethemoglobin method [29], packed cell volume (PCV) with Wintrobe Hematocrit tube method [30], total erythrocyte counts (TEC), and total leukocyte counts (TLC) using a hemocytometer [31], and differential leukocyte counts (DLC) with a standard hematological procedure using Leishman stain (Hi-media Laboratories Pvt. Ltd., Thane, India) were measured [31]. Blood indices were calculated from the obtained results using the specific formulas, viz., mean corpuscular volume (MCV, in femtoliter) = PCV% × 10/TEC number (millions/mm^3^), mean corpuscular Hb (MCH, in picograms) = Hb (g/dL) × 10/TEC number (millions/mm^3^), and mean corpuscular Hb concentration (MCHC, in g/dL) = Hb (g/dL) × 100/PCV (%).

Blood biochemical variables, viz., glucose, total protein, albumin, total cholesterol and creatinine, and enzymes, viz., alkaline phosphatase (ALP), aspartate transaminase (AST), and alanine transaminase (ALT), were analyzed from plasma using commercially available kits (Transasia, Bio-Medicals Ltd., Mumbai, India) as per manufacturer’s protocol.

Endocrines, viz., cortisol, T_3_, and T_4_, were determined using commercially available ELISA kits following the manufacturer’s protocol (DRG Diagnostics, Marburg, Germany).

#### 2.2.4. Fecal Oocyst and Microbial Count, Phagocytic Activity, and Lymphocyte Proliferation Response

Oocyst output was measured in terms of oocysts per gram (OPG) of feces with the McMaster method [32] at weekly intervals on 7, 14, and 21 DPI. The samples were collected from two birds per replicate (*n* = 2 per replicate) across six experimental groups. The cloacal swabs were used to quantify *Escherichia coli*, *Salmonella* spp., and *Lactobacillus* spp. numbers with spread plate method [33] using species-specific media on 7, 14, and 21 DPI from four birds in each replicate across six experimental groups. Phagocytic activity of neutrophils was measured [34] using nitroblue tetrazolium (Hi-media Laboratories Pvt. Ltd., Thane, India), and lymphocyte proliferation response was determined [35] using the colorimetric MTT (3-(4,5-dimethylthiazolyl-2)-2,5-diphenyltetrazolium bromide) (Hi-media Laboratories Pvt. Ltd., Thane, India) on 7, 14, and 21 DPI in four birds per replicate for each group.

#### 2.2.5. Preparation of Crude Somatic Antigen of *Eimeria* Species and Antibody Titer Measurement

Cecal contents were collected from the *Eimeria*-infected slaughtered birds on 7, 14, and 21 DPI from two birds per replicate (*n* = 2/replicates) across six experimental groups and the oocysts were separated using the standard sedimentation and centrifugal flotation technique [32]. The separated oocysts were sporulated using 2.5% potassium dichromate following the standard method [32]. After washing with phosphate buffer solution, the oocysts were disintegrated by vortexing with glass beads (0.05 mm size) and sonicated in an ultrasound homogenizer (BANDELIN electronic GmbH & Co. KG, Berlin, Germany) using proteinase K (Qiagen, Hilden, Germany). The sonicated material was centrifuged in a cold centrifuge (Hermle, Reichenbach, Germany). The supernatant was collected as a somatic antigen of *Eimeria* species and protein content was measured [36].

Immunoglobulin Y (IgY) antibody titer against the somatic antigen of *Eimeria* spp. was measured in the serum of experimentally infected birds with indirect ELISA (i-ELISA) method [37] on 7, 14, and 21 DPI in four birds per replicate for each group. Serial dilution of serum was prepared up to five times starting from 1:100 dilutions. The crude somatic antigen of *Eimeria* spp. (5 µg/well) was used as a coating antigen and rabbit anti-chicken IgY-HRPO (HiMedia, Thane, India) was used as the conjugated secondary antibody. The sera from the infected control groups and healthy control group were considered positive control and negative control, respectively. The absorbance of the wells was measured at 492 nm in an ELISA Reader (Thermo Electron Corporation, Thermo Fisher Scientific, Waltham, MA, USA).

#### 2.2.6. Gene Expressions

Fresh tissue samples from the jejunum were collected on 7 and 21 DPI from one bird of each replicate and immersed in RNA-later solution (Thermo Fisher Scientific, Waltham, MA, USA) and stored at −80 °C until used for determining the expression of interleukin 10 (*IL-10*), interferon-gamma (*IFN-γ*), and Toll-like receptor 4 (*TLR-4*) genes. Briefly, each tissue sample was homogenized separately in a Polytron homogenizer for RNA extraction. After homogenization, the sample was transferred to a 2.0 mL centrifuge tube, and total RNA was extracted using Tri reagent (Sigma-Aldrich, St. Louis, MO, USA) following the manufacturer’s instructions. The integrity of the total RNA was checked by performing agarose gel electrophoresis through the Gel documentation system (Bio-Rad Laboratories Inc., Hercules, CA, USA) and total RNA was quantified using a Nanodrop spectrophotometer (Eppendorf, Hamburg, Germany). Total RNA was treated with RNase-free DNAseI (Fermentas Life Sciences, Burlington, ON, Canada) to exclude genomic DNA contamination. Then, iScript Reverse Transcriptase cDNA synthesis kit (Bio-Rad Laboratories Inc., Hercules, CA, USA) was used for synthesizing cDNA. Quantification of mRNA level was achieved via quantitative real-time polymerase chain reaction (qPCR) (CFX96, Bio-Rad Laboratories Inc., Hercules, CA, USA) using the primers specific to *IL-10*, *IFN-γ*, and *TLR-4* genes along with the housekeeping gene (*GAPDH*) (Supplementary Table S1) [38]. The gene expression levels were calculated [39] relative to the gene expression in NC at 0 DPI.

### 2.3. Statistical Analysis

Data were analyzed using PROC MIXED procedures of SAS [40], and the model contained treatment, week/DPI, and treatment × week interactions as main effects and pen/bird as a random effect for all variables except for antibody titers using the following statistical model:

Y*_ijk_* = μ + T*_i_* + W*_j_* + (T × W)*_ij_* + a*_k_* + e*_ijk_*

where Y*_ijkl_* = dependent variable, μ = overall mean, T*_i_* = effect of treatment *i*, W*_j_* = effect of week *_j_*, (T × W)*_ij_* = interaction effect of treatment *i* and week *j*, a*_k_* = random effect of pen or birds *k*, and e*_ijk_* = overall residual error.

The model for antibody titer included treatment, DPI, dilution, treatment × DPI, and treatment × DPI × dilution as main effects. When an interaction effect was significant (*p* < 0.05), the ‘SLICE’ option in the SAS model was used to find the significant difference (*p* < 0.05) among treatments within a week/DPI or among DPI within a treatment. Subsequently, significant differences (*p* < 0.05) among the treatments within a week/DPI or among the weeks/DPIs within a treatment were detected using pairwise comparisons using Fisher’s protected least significant difference test. Microbial and oocyst counts were log10-transformed before statistical analysis. Gene expression data were log2-transformed before analysis and then back-transformed to present in the table. Statistical significance was set at *p* ≤ 0.05.

## 3. Results

### 3.1. Growth Performances

No mortality was recorded in any group. Among the various growth and feed utilization attributes, only ADG and ADFI differed (*p* < 0.05) across the treatments (Trt). All the attributes increased (*p* < 0.01) with age and were influenced (*p* < 0.01) by the interactions between Trt and DPI (Table 3). After five weeks, the BA and Sal groups exhibited higher BW and ADG than the control (NC) and PC groups, followed by the OEO and B&O groups. At the end of this study, the B&O and OEO groups had the lowest ADFI and FCR, followed by the BA and Sal groups. Throughout the trial (0–5 weeks), the broiler chickens supplemented with OEO, B&O, and BA showed the best growth performance and feed consumption, outperforming the NC group and showing comparable results to the Sal group.

### 3.2. Hematological Features

There was no (*p* > 0.05) treatment effect on Hb, PCV, TEC, MCV, MCH, MCHC, or TLC among the groups (Table 4). Except for MCHC, significant differences (*p* < 0.05) among various DPI were observed in all other variables (*p* < 0.01). The interaction between Trt and DPI had a strong impact on Hb (*p* < 0.01), MCHC (*p* < 0.01), and MCH (*p* < 0.05). After the seventh DPI, the Hb concentration decreased, particularly in the PC and Sal groups. The PCV increased starting from 14 DPI. At this time (14 DPI), the TEC decreased (*p* < 0.05), while the MCV and MCH increased (*p* < 0.05) for all groups. After 7 DPI, the MCH level rose in the Sal and PC groups. There was no notable difference (*p* > 0.05) in MCHC among the groups or DPI. At 14 DPI, there was a decrease (*p* < 0.05) in TLC. Except for monocyte counts (%), which dropped at 21 DPI, various leukocyte counts including the heterophil to lymphocyte (H/L) ratio did not differ (*p* > 0.05) among the treatment groups, DPI, or Trt × DPI interactions (Table 5).

### 3.3. Enzymes, Endocrines and Biochemical Profile

The concentrations of endocrines and liver enzyme profiles were similar (*p* > 0.05) among the treatment groups (Table 6). As DPI and age progressed, a decrease (*p* < 0.01) in ALP and an increase in T_3_ levels were observed across the groups, with the cortisol concentration dropping from 14 DPI. The only significant (*p* = 0.035) interaction between Trt and DPI was found for T_3_ levels. At 7 DPI, the Sal group showed a lower (*p* < 0.05) T_3_ level than the other groups. Except for creatinine concentration and the albumin-to-globulin ratio, all biochemical profiles displayed significant changes (*p* < 0.05) among DPIs, while the treatment and Trt × DPI had no effect (*p* > 0.05) (Table 7). Plasma glucose concentration decreased during mid-infection; however, total protein, albumin, globulin, and cholesterol concentration increased at 14 DPI.

### 3.4. Fecal Oocyst and Microbial Count, Phagocytic Activity and Lymphocyte Proliferation Response

No *Eimeria* spp. oocysts were found in the negative control group during the study. Fecal OPG counts were higher (*p* < 0.01) in the PC group than in all the supplemented groups and declined (*p* < 0.01) after 14 DPI (Table 8). All the bacterial counts were altered (*p* < 0.01) by the group, DPI, and Trt × DPI interactions throughout the post-infection period, except for the non-significant (*p* > 0.05) effect of DPI on *Lactobacillus* (Table 8). The overall *Salmonella* and *E. coli* counts were highest in the PC group and lowest in the B&O group, respectively, and they followed the following pattern: B&O < OEO < BA < Sal, NC < PC and B&O < OEO, BA < Sal, NC < PC, respectively, whereas the *Lactobacillus* count showed the opposite pattern: B&O > OEO > BA > Sal > NC > PC. As age progressed, the number of *E. coli* increased while the number of *Salmonella* declined gradually, a pattern consistent across all groups at each DPI. The phagocytic activity of heterophils remained constant (*p* > 0.05) across groups and Trt × DPI interactions; however, phagocytic activity increased (*p* < 0.01) as DPI advanced (Table 8). There was a difference (*p* < 0.01) in the lymphocyte proliferation response between the B&O and PC groups, with the B&O showing the highest response and the PC the lowest (B&O > PC > BA, OEO, Sal, and NC). DPI had no effect (*p* > 0.05) on lymphocyte proliferation response (Table 8).

### 3.5. Serum IgY Titer

**Figure 1:**
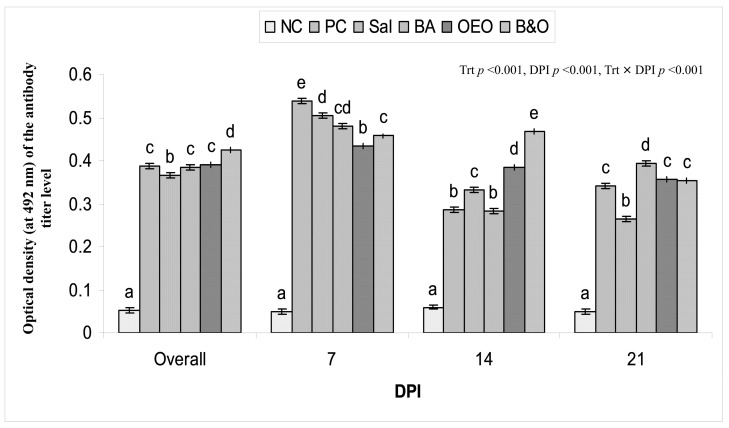
Optical density (at 492 nm) of the antibody titer level in diluted serum of broiler chickens supplemented with different anticoccidial agents and challenged with coccidia. NC = negative control chickens, PC = positive control chickens, coccidia-challenged and non-treated, Sal = coccidia-challenged chickens + salinomycin at 60 mg/kg of feed, BA = coccidia-challenged chickens + benzoic acid at 500 mg/kg of feed, OEO = coccidia-challenged + oregano essential oil at 500 mg/kg of feed, B&O = coccidia-challenged + benzoic acid at 500 mg/kg of feed + oregano essential oil at 500 mg/kg of feed, Trt = treatment, DPI = days post-infection. ^a,b,c,d,e^ Means with different letters for each column bar differ significantly (*p* < 0.05) among the treatments (Fisher’s protected least significant difference test).

*Eimeria*-specific IgY levels, in terms of optical density at 492 nm, were detected up to a maximum of four times the dilution (D4; 1:800) in all the groups and were affected (*p* < 0.01) by treatment, dilutions (Dil), DPI, Trt × DPI interactions, and Trt × DPI × Dil interactions (Figure 1). The overall *Eimeria*-specific IgY response of different groups ranged from 0.053 to 0.426 OD, with the highest response observed in the B&O group, followed by the OEO, BA, PC, and Sal groups. The NC group had the lowest IgY level. At 7 DPI, the IgY titer responded more strongly, reaching 0.411 OD. Subsequently, the levels decreased, reaching a range of 0.293 to 0.303 OD at 21 DPI. On 7 DPI, the IgY titer was highest in the *Eimeria*-challenged, non-supplemented group, followed by supplemented groups with the following order: PC > Sal, BA > B&O > OEO > NC. On 14 DPI, the IgY titer altered in different groups with the following order: B&O > OEO > Sal > BA, PC > NC and on 21 DPI, BA > B&O, OEO, PC > Sal > NC. The titer in the BA group remained stable throughout the duration of post-infection; however, it gradually decreased in the Sal and PC groups while showing improvement in the B&O and OEO groups as the DPI progressed.

### 3.6. Relative Expression of Interleukin 10, Interferon Gamma and Toll-like Receptor 4 Genes in Jejunum

**Figure 2:**
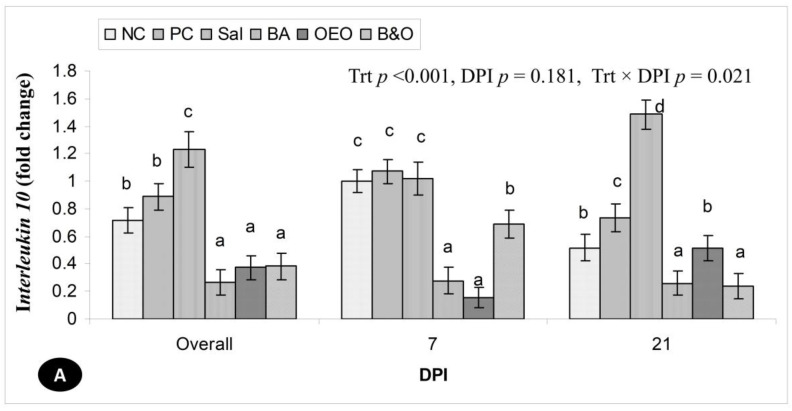

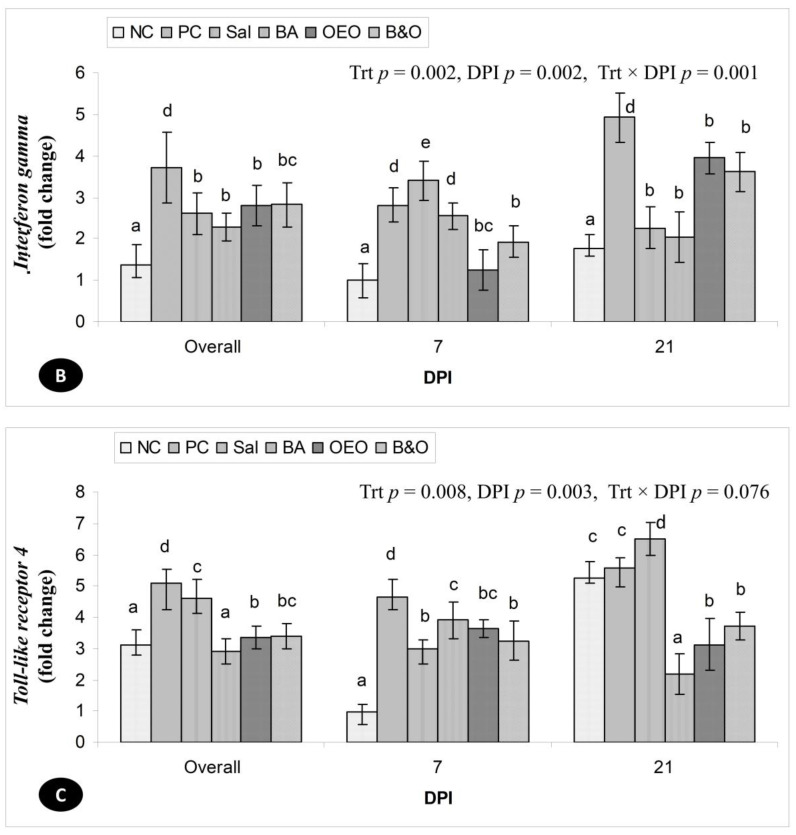
Relative expression (fold change) of (**A**) interleukin 10, (**B**) interferon gamma, and (**C**) Toll-like receptor 4 genes in the jejunum of broiler chickens supplemented with different anticoccidial agents and challenged with coccidia. NC = negative control chickens, PC = positive control chickens, coccidia-challenged and non-treated, Sal = coccidia-challenged chickens + salinomycin at 60 mg/kg of feed, BA = coccidia-challenged chickens + benzoic acid at 500 mg/kg of feed, OEO = coccidia-challenged + oregano essential oil at 500 mg/kg of feed, B&O = coccidia-challenged + benzoic acid at 500 mg/kg of feed + oregano essential oil at 500 mg/kg of feed, DPI = days post-infection. ^a,b,c,d,e^ Means with different letters for each column bar differ significantly (*p* < 0.05) among the treatments (Fisher’s protected least significant difference test).

The relative expression of the *IL-10* gene was highest (*p* < 0.01) in the Sal group, followed by the PC and NC groups, with lower expression observed in the B&O, OEO, and BA groups (Figure 2A). Although there was no difference in the relative expression of *IL-10* between the DPIs, the Sal and PC groups exhibited higher expression levels, and the BA group showed lower expression in both DPIs. In the B&O and NC groups, the expression was higher at 21 DPI, whereas it was lower in the OEO group. The overall relative expression of the *IFN-γ* gene showed the highest (*p* < 0.01) level in the PC group, followed by B&O and OEO, then the Sal and BA groups, while the lowest was found in the NC group (Figure 2B). The relative expression of *IFN-γ* increased (*p* < 0.01) progressively in the B&O, OEO, and PC groups with the time following coccidia challenge; in contrast, it decreased in the Sal and BA groups and was unchanged in the NC group. The overall relative expression of the *TLR-4* gene was highest (*p* < 0.01) in the PC group, followed by the Sal, B&O, and OEO groups, and was lowest in the BA and NC groups (Figure 2C). The relative expression of *TLR-4* increased (*p* < 0.01) progressively as DPI increased in the NC, PC, and Sal groups but it decreased in the BA and OEO groups and did not change in the B&O group.

## 4. Discussion

### 4.1. Growth Performances

The higher BW and ADG in the Sal and BA groups might be due to better gut morphology and a reduced pathogenic gut-microbial population [23]. A favorable gut environment for nutrient utilization may stimulate appetite, thus resulting in a modest increase in ADFI and a decrease in FCR in the salinomycin-supplemented group [41] and BA-added group [42]. Dietary essential oils might enhance the gut-beneficial microbiota population, reduce the oocyst population, and improve gut histomorphology [22,43], resulting in better nutrient efficiency and ADG in the OEO and B&O groups of birds. The combined effect of organic acid (OA) and essential oils (EO) might improve growth performance due to better nutrient digestibility and gut health of *Eimeria*-challenged broilers [44] by stimulating endogenous digestive enzyme, bile, and mucus secretion [45]. Increased feed efficiency was also reported as a synergistic effect of OA + EO increasing *Lactobacillus* and decreasing pathogenic bacteria populations in birds [15]. In this current study, the low growth performance in the PC group might be due to *Eimeria* infection followed by other pathogenic bacteria in the gut [16,46] that impair the gut microstructures of chickens [47] leading to impaired digestion and nutrient absorption with higher ADFI and FCR [48].

### 4.2. Hematological Features

The lowest Hb concentration in PC groups at 7 DPI may be due to the mechanical disruption of cecal mucosal capillaries caused by the *Eimeria* schizogony contamination, causing hemorrhages [20]. A similar effect on Hb and MCH concentrations was found in the Sal group due to the acute phase of *Eimeria* infection [49]. A steady Hb concentration might occur in coccidia-challenged birds due to reduced hemorrhage caused by supplementation of OEO [20] and organic acid in the BA group [10]. The reduction of Hb levels in the non-infected group was unexpected and might have no relation to the study. The initial significant reduction in PCV and MCV at 7 DPI irrespective of group might be due to body fluid alteration during acute infectivity [49]. These values increased at the recovery phase from 14 DPI, which might be due to the age effect and occurrence of large-sized immature RBCs after hemorrhage [50]. The reduced levels of TEC in 14 DPI may be due to the post-effect of acute coccidial infection beyond 7 DPI [51], which was restored within a week at 21 DPI by a compensatory mechanism of erythropoiesis against blood loss during infection [52]. The pulsate changes in leukocyte count might be due to the initiation of immune reaction followed by reduced inflammatory response that leads to subsequent normalization in coccidiosis [53]. This study showed that all infected birds maintained hematological values within the normal physiological range [49].

The non-significant alteration of heterophil, basophil, lymphocyte and monocyte percentage, and H/L ratio in *coccidia*-challenged and non-challenged broiler chickens in different DPIs might be due to less stress in the developed coccidiosis in this present study [54]. The monocytes might be involved efficiently in *Eimeria* infection as a part of the host-defense mechanism [49] to remove necrotic cells and tissue debris invading microorganisms, a major phagocytic component [9]. The subsequent decrease of monocytes during the later stage indicated the resolution of the initial immune response [55].

### 4.3. Enzymes, Endocrines, and Biochemical Profiles

The non-significant alteration of AST, ALT, and ALP among the groups indicated that the supplementation of anticoccidials and coccidia challenge had no hepatotoxic effects on the chickens [54]. The activity of ALP gradually decreased with increasing DPI, which may be due to increased osteoblastic activity and mineralization to boost the skeletal growth in the growing phase of young birds [56].

The overall T_3_ and T_4_ levels of different groups were found to be lower than the findings of Moryani et al. [57], and the cortisol level was found to be in line with the reported ranges of Farahani and Hosseinian [58] in broiler chickens. T_3_ synthesis is stimulated by healthy gut microbiota [59] that increase with age for optimum BW gain [60]. In this present study, the T_3_ level had a positive correlation with *E. coli* population [61]. The role of salinomycin in T_3_ synthesis in chickens has not been discussed due to the paucity of the literature; hence, the cause of decreased T_3_ level only at 7 DPI in the Sal group remained unexplained. However, it was reported in sheep that salinomycin had no significant effect on plasma T_3_ levels [62]. A constant level of thyroxine secretion in this present study might be controlled by the dogmatic secretion of a thyrotropin-releasing hormone from the hypothalamus influenced by the overlapping role of T_3_ and cortisol to encompass a homeostatic mechanism [63,64]. *Coccidia* may exacerbate lipid peroxidation and increase oxidative stress along with elevated cortisol levels on the 7 DPI. This condition likely improves as the infection progresses, possibly due to a decrease in coccidia numbers [65]. Birds gradually adapted to the stressors from *Eimeria* infection and may have returned to baseline cortisol levels over time [66]. Therefore, it can be concluded that the physiological concentration of the triiodothyronine (T_3_) hormone increased with age in broiler chickens, likely due to the influence of their gut microbiome and the reduction of *Eimeria*-infective stress, which down-regulated cortisol levels, and the commercial anticoccidial salinomycin was found to lower T_3_ levels.

Infection-induced cortisol might cause an increase in glucose levels during the early stages of infection [67]. Gluconeogenesis may be inhibited by intestinal tract inflammation at the recovery phase in 14 DPI [65], followed by the mobilization of organic reserves to restore the insufficiency in hepatic glycogen caused by higher glucose after the recovery phase in broiler chickens [68]. The periodic alteration of the total protein and its components could be related to coccidiosis [1] and time-dependent alterations of protein catabolism related to growth and fattening in broiler chickens [69]. Alteration in cholesterol levels may be due to inflammation leading to the redistribution of lipids and lipoproteins [70].

### 4.4. Fecal Oocyst and Microbial Count, Phagocytic Activity, and Lymphocyte Proliferation Response

*Eimeria* oocyst shedding in the excreta is considered the chief determining factor concerning the intensity of coccidial infection and is potentially linked to ADG and FCR [71]. The higher oocyst shedding continued up to their merogony stage until 14 DPI and decreased gradually due to the intrinsic decline of merozoites [4,10]. Salinomycin causes the movement of cations across membranes with an influx of sodium and calcium, and an efflux of potassium resulting in changes in pH and metabolic processes within *Eimeria* [72]. The disruption of ion gradients across the cell membranes might lead to the suppression of *Eimeria* oocysts in feces [45]. Oregano essential oil might discompose the cellular membrane by its active compounds (thymol and carvacrol), inhibit ATPase activity, and release intracellular ATP of the *Eimeria*, followed by interference of the ionic exchange leading to death of the coccidia [73]. This present study supported that essential oils may also be effective in reducing the coccidia life cycle in naturally occurring environmental coccidiosis under free-range breeding systems [74]. Benzoic acid, like other organic acids, could reduce the OPG count by inhibiting oxidative phosphorylation or electron transport to the coccidia and subsequently disturbing the life cycle of *Eimeria* [10].

The gradual increase in *E. coli* count might be the consequence of *E. tenella* infection, which might favor the proliferation and survivability of pathogens [47] secreting *IFN-γ* and disrupting heterophil extracellular traps [75], and is a common occurrence of progression of bird’s age [76]. An initial higher count of *Salmonella* might be the cause of *Eimeria* infection [47] that decreased with the advancement of DPI and is associated with the higher population of beneficial commensal bacteria, such as *Lactobacillus* preventing colonization of *Salmonella* through competitive exclusion [77]. Higher *E. coli* and *Salmonella* counts were detected in the *Eimeria*-challenged PC chickens, which could be due to the creation of wounds in the intestinal epithelium initiated by *E. tenella* favoring the proliferation and survivability of gut pathogenic microbial populations [47] utilizing the cytokines, such as *IL-10* and *IFN-γ*, and some Toll-like receptors [78]. The supplementation of OEO might reduce *E. coli* and *Salmonella* spp. [79], disrupting their cell membranes by secretion of reactive oxygen species [80]. The process is activated by cytokines like *IFN-γ* in chickens [81], causing lower *E. coli* and *Salmonella* counts in the OEO and B&O groups. Reduced levels of *E. coli* and *Salmonella* spp. in BA-supplemented coccidia-challenged chickens in this present study as well as an earlier study by Zhang et al. [13] might be attributed to a lowering effect of the bacterial intracellular pH by uncoupling the electron transport, leading to the interference of the cytoplasmic membrane structure and membrane proteins [81].

Benzoic acid reduces intestinal pH, which might facilitate thereduction of the *Eimeria* spp. [11] and favor *Lactobacillus* growth [81]. The blending effect of OEO and BA indicated a positive effect on gut health by declining *Eimeria* spp. and amplifying *Lactobacillus* spp. in broiler chickens [82,83]. The present findings signify that the combination of OEO and BA can reduce the harmful Gram-negative microbiota and stimulate the growth of beneficial microflora in broiler chickens [84] compared with the salinomycin-treated group.

Phagocytic activity was modified with age [85]. During infections, heterophils are primed by *IFN-γ*, which promotes their binding with *TLR-4* [86], which leads to higher phagocytic activity with the advancement of infection [87]. Higher free radical generation in response to *Eimeria* was reported earlier in mice [88], which may explain higher phagocytic activity at 7 DPI. The lymphocyte proliferation response involving T-cells in protective immunity against *coccidia* has been recognized to reduce oocyte excretion [89]. The infection with *Eimeria* species might activate antigen-specific T-cell-induced immune response [90], causing higher T lymphocyte proliferation in all the *Eimeria*-challenged groups. Both OEO and BA are potential antimicrobial agents, having effective immunomodulatory properties [17], and might induce lymphocyte proliferation when used alone or in combination [14]. Salinomycin might induce T-cell proliferation by inhibiting the expression and enzymatic activity of immunosuppressive indoleamine-2,3-dioxygenase [91]. Hence, the steady activation of heterophils in terms of phagocytic activity potentially boosted the immune system in the experimental chickens and had a stable dominating role of lymphocyte proliferation response in the B&O group, followed by their individual supplemented groups (BA and OEO), which resisted the coccidiosis satisfactorily.

### 4.5. Antibody Titer (Serum IgY)

A higher level of IgY in the PC group initially at 7 DPI might be due to the natural resistance to *Eimeria* developed by invading the host’s intestinal epithelial cells, which induced the production of specific antibodies owing to the excess occurrence of the sporozoites and merozoites to facilitate a stronger antibody titer [92]. The IgY level declined from 14 DPI probably due to the reduced replication and maturation of *Eimeria* resulting in a lower secretion of specific antibodies [92]. The IgY potentially overwhelmed the host’s immune defenses and gradually weakened with the adaptation of the parasite, leading to a decline in antibody titers 21 DPI [93]. BA could improve the humoral immunity against *Eimeria*-infected broilers by raising the *Lactobacillus* population in the gut [81], which modulated the host immune response and improved antibody production [94]. The immune-stimulating effect of OEO might influence humoral immunity along with the mononuclear phagocyte and cellular immune systems in chickens, leading to better IgY in the OEO and B&O groups [95]. The gradual decline of IgY levels with the advancement of infection in the salinomycin-supplemented chickens could be indicative of the development of resistance to the drug or its inability to stimulate the host immune response over time [96]. The presence of IgY in the non-infected NC group could possibly be the natural antibody produced at baseline levels as a part of the immune system to provide passive immunity transferred from maternal antibodies [93]. Hence, it can be concluded that the *Eimeria*-specific IgY titer in the infected chickens reduced after 7 DPI and OEO supplementation either alone or in combination with the BA could improve it compared with the salinomycin-supplemented chickens.

### 4.6. Immune Gene Expressions

*Eimeria* spp. may induce the secretion of *IL-10* to ease their invasion into chicken epithelial cells and reduce the pro-inflammatory *IFN-γ*, and thus, the down-regulation of *IL-10* has a significant role in minimizing inflammatory responses [97]. A similar effect was noticed in the OEO and B&O groups but not in the Sal and PC groups. The OEO has an anti-inflammatory effect and down-regulates *IL-10* expression in the birds, acting as a possible antibiotic substitute to control coccidiosis [25]. The Sal group had a lower expression of *IL-10* and a greater expression of *IFN-γ* compared with the PC, contradicting the findings of Lee et al. [98]. An early and modest induction of *IL-10* might occur in the non-infected NC group under normal homeostatic conditions in the jejunum that did not negatively impact resistance to *Eimeria*-infection [99].

The expression of *IFN-γ* increased in the OEO and B&O groups, which, along with the anti-inflammatory effect, helped to minimize the pathogenic effect of coccidia [100]. In the BA group, higher *IFN-γ* expression might be due to the dietary supplementation of organic acid [101], which probably influenced the *Lactobacillus* population to stimulate *IFN-γ* production [102]. This present study demonstrated that IFN-γ is one of the cytokines involved in coccidiosis that directly inhibits the development of *Eimeria* spp. within the cells [97].

TLR-4 is a transmembrane protein involved in developing specific adaptive immunity in chickens induced by *Eimeria* infection to promote an inflammatory response [103]. Thus, *TLR-4* expressions increased in the PC group in this present study. However, *TLR-4* might also respond to *E. coli* and *Salmonella* infection [9]. TLR functions can be modified by dietary manipulation (e.g., OEO) to downregulate the *TLR-4*-mediated inflammatory pathways, causing a lowered expression of it in the OEO and B&O groups than in the Sal and PC groups [25]. This present study signifies that BA and OEO alone and in combination increased the expression of *IFN-γ* and reduced *IL-10* and *TLR-4* to protect the chickens from redundant inflammatory reactions that were not observed in salinomycin-treated birds.

## 5. Conclusions

The combined dietary supplementation of BA and OEO in chickens prevented coccidiosis largely by decreasing the OPG count, increasing *Eimeria*-specific IgY production, and improving immune responses and FCR. OEO-supplemented chickens accomplished better ADG, and in combination with BA, increased the beneficial *Lactobacillus* population and reduced harmful gut *E. coli* and *Salmonella*. Salinomycin lowered the T_3_ level at the initial phase of *Eimeria* infection. Thus, the combined application of OEO and BA may substitute for an anti-coccidial agent, salinomycin, in controlling coccidiosis with antibiotic-free animal food products. This may reduce the environmental burden of pathogenic antimicrobial resistance.

## Figures and Tables

**Table 1:** Description of the experimental design.

Group	NC	PC	Sal	BA	OEO	B&O
Birds	Six replicates with seven birds in each	Six replicates with seven birds in each	Six replicates with seven birds in each	Six replicates with seven birds in each	Six replicates with seven birds in each	Six replicates with seven birds in each
Group description	Non-challenged, no additive	Coccidia-challenged, no additive	Anti-coccidial drug + coccidia-challenged	BA + coccidia-challenged	OEO + coccidia-challenged	BA + OEO + coccidia-challenged
Day 1 to 35	No additive	No additive	Salinomycin at 60 mg/kg feed	BA at 0.50 g/kg feed	OEO at 0.50 g/kg feed	BA at 0.50 g/kg of feed + OEO at 0.5 g/kg feed
Day—14 (0 DPI)	No coccidial challenge	Challenged with coccidia	Challenged with coccidia	Challenged with coccidia	Challenged with coccidia	Challenged with coccidia

NC = Negative control, PC = Positive control, Sal = Salinomycin, BA = Benzoic acid, OEO = Oregano essential oil, B&O = Benzoic acid and oregano essential oil, DPI = days post-infection.

**Table 2:** Ingredient and nutrient composition of the diets fed to broiler chickens in different periods.

	Starter (Day 1–14)	Grower (Day 15–28)	Finisher (Day 29–35)
Ingredient composition (g/kg)			
Maize	565	580	600
Vegetable oil	15	20	30
Soybean (45% crude protein)	380	360	330
Salt	2.5	2.5	2.5
Dicalcium phosphate	14	14	14
Limestone powder	10	10	10
DL-Methionine	2.8	2.8	2.8
L-Lysine HCl	2.7	2.7	2.7
L-Threonine	0.7	0.7	0.7
Vitamin and min premix ^1^	1.9	1.9	1.9
Other premix ^2^	5.4	5.4	5.4
Nutrient composition g/kg, on dry matter basis) ^3^			
Dry matter	898	900	899
Crude protein	224	202	195
Ether extract	33	34.7	49.9
Crude fibre	31.7	31.6	31.9
Total ash	51.0	55.4	55.6
Acid insoluble ash	23.2	27.7	23.3
Calcium	9.2	8.9	8.6
Total phosphorus	7.4	7.3	7.7
Metabolizable energy (Kcal/kg)	3007	3066	3175
Lysine	14.0	12.3	11.2
Methionine	6.2	5.3	5.6
Cystine	3.7	3.2	3.3
Methionine and cystine	9.96	9.23	9.07
Arginine	16.2	14.2	13.4
Tryptophan	2.5	2.5	2.5
Threonine	8.6	7.8	7.4

^1^ The supplemented mixture of minerals and vitamins manufactured by Rik Feed, Kolkata, India, provided with retinyl acetate 3.75 mg, 1,25-di-hydroxy-cholecalciferol 4 mg, DL α-tocopheryl acetate 30 mg, menadione 4 mg, thiamine propyldisulfide 3 mg, riboflavin tetrabutyrate 8 mg, methyl cobalamine 0.025 mg, sodium pantothenate 15 mg, pyridoxine 5 mg, niacin 60 mg, biotin 0.2 mg, folic acid 2 mg, manganese 40 mg, iron 30 mg, zinc 25 mg, copper 3.5 mg, iodine 0.3 mg, selenium 0.15 mg, choline chloride 200 mg per kilogram of feed. ^2^ Other feed additives contained phytase 5000, choline chloride, toxin binder, cocktail enzyme, cocciostat, emulsifier, antioxidant, sodium bicarbonate, and liver tonic manufactured by Rik Feed, Kolkata, West Bengal, India. ^3^ The mentioned nutrient level was provided by the manufacturer and presented in the table.

**Table 3:** Growth performances of broiler chickens supplemented with different anticoccidial agents and challenged with coccidia.

Variables	Week	Treatment (Trt)						Week_m_	SEM	*p*-Value		
		NC	PC	Sal	BA	OEO	B&O			Trt	DPI	Trt × DPI
BW (g)	1	205 ^m^	202 ^m^	203 ^m^	206 ^m^	204 ^m^	202 ^m^	204 ^m^	24.3	0.371	<0.001	<0.001
	2	467 ^n^	502 ^n^	533 ^n^	520 ^n^	510 ^n^	511 ^n^	507 ^n^				
	3	950 ^o^	965 ^o^	1021 ^o^	994 ^o^	988 ^o^	969 ^o^	981 ^o^				
	4	1561 ^p^	1528 ^p^	1611 ^p^	1580 ^p^	1549 ^p^	1559 ^p^	1565 ^p^				
	5	2106 _a_^q^	2073 _a_^q^	2228 _c_^q^	2213 _c_^q^	2136 _b_^q^	2143 _b_^q^	2150 ^q^				
ADG (g/d)	0–1	51.8 _b_^n^	26.1 _a_^m^	25.8 _a_^m^	28.1 _a_^m^	27.7 _a_^m^	26.7 _a_^m^	31.0 ^m^	1.34	0.041	<0.001	<0.001
	1–2	37.6 ^m^	42.9 ^n^	47.1 ^n^	44.8 ^n^	43.7 ^n^	44.1 ^n^	43.4 ^n^				
	2–3	68.9 ^o^	66.2 ^o^	69.8 ^o^	67.7 ^o^	68.3 ^o^	68.5 ^o^	67.7 ^o^				
	3–4	87.3 ^q^	80.5 ^p^	81.8 ^p^	83.8 ^p^	80.1 ^p^	81.1 ^p^	82.5 ^p^				
	4–5	77.9 _a_^p^	77.7 _a_^p^	88.1 _b_^p^	90.4 _b_^p^	83.9 _ab_^p^	83.5 _ab_^p^	83.6 ^q^				
	0–5	64.7 _b_	58.7 _a_	62.5 _ab_	63.0 _b_	60.7 _a_	60.2 _a_					
ADFI (g/d)	0–1	48.1 _b_^m^	29.6 _a_^m^	31.1 _a_^m^	31.3 _a_^m^	30.3 _a_^m^	29.9 _a_^m^	33.4	1.65	0.017	<0.001	0.002
	1–2	42.1 ^m^	46.7 ^n^	51.2 ^n^	48.6 _n_	47.5 ^n^	48.2 ^n^	47.4 ^n^				
	2–3	92.3 ^n^	84.44 ^o^	93.51 ^o^	89.15 ^o^	89.53 ^o^	86.91 ^o^	89.30 ^o^				
	3–4	123 ^o^	119 ^p^	124 ^p^	122 ^p^	121 ^p^	120 ^p^	121 ^p^				
	4–5	140 _ab_^p^	133 _a_^q^	144 _b_^q^	141 _b_^q^	129 _a_^q^	128 _a_^q^	136 ^q^				
	0–5	89.1 _b_	82.5 _a_	88.9 _b_	86.5 _ab_	83.4 _a_	82.6 _a_					
FCR	0–1	1.01 _a_^m^	1.14 _bc_^m^	1.21 _b_^n^	1.11 _bc_^m^	1.10 _ab_^m^	1.12 _b_^m^	1.12 ^m^	0.019	0.337	<0.001	<0.001
	1–2	1.11 ^n^	1.09 ^m^	1.09 ^m^	1.09 ^m^	1.09 ^m^	1.09 ^m^	1.09 ^m^				
	2–3	1.34 ^n^	1.28 ^n^	1.34 ^o^	1.31 ^n^	1.31 ^n^	1.33 ^n^	1.32 ^n^				
	3–4	1.41 n	1.47 ^o^	1.52 ^p^	1.46 ^o^	1.51 ^o^	1.49 ^o^	1.48 ^o^				
	4–5	1.80 _d_^o^	1.71 _cd_^p^	1.64 _bc_^q^	1.56 _ab_^o^	1.54 _ab_^o^	1.53 _a_^o^	1.63 ^p^				
	0–5	1.33	1.34	1.36	1.31	1.31	1.31					

BW = body weight, ADG = average daily body weight gain, ADFI = average daily feed intake, FCR = feed conversion ratio (feed/gain), NC = negative control chickens, PC = positive control chickens, coccidia-challenged and non-treated, Sal = coccidia-challenged chickens + salinomycin at 60 mg/kg of feed, BA = coccidia-challenged chickens + benzoic acid at 500 mg/kg of feed, OEO = coccidia-challenged+ oregano essential oil at 500 mg/kg of feed, B&O = coccidia-challenged + benzoic acid at 500 mg/kg of feed + oregano essential oil at 500 mg/kg of feed. Week_m_ = Mean of the Week, SEM = Pooled standard error of mean. ^a,b,c,d^ Means with different subscript letters within a row differ significantly (*p* < 0.05) among the treatments. ^m,n,o,p,q^ Means with different superscript letters within a column differ significantly (*p* < 0.05) among the weeks/DPI (Fisher’s protected least significant difference test).

**Table 4:** Hematological features of broiler chickens supplemented with different anticoccidial agents and challenged with coccidia.

Variables	DPI	Treatment (Trt)						DPI_m_	SEM	*p*-Value		
		NC	PC	Sal	BA	OEO	B&O			Trt	DPI	Trt × DPI ^1^
Hb (g/dL)	Overall	10.4	9.40	9.69	10.2	10.3	9.84		0.47	0.556	0.022	0.006
	7	11.2 ^y^	7.38 ^x^	7.55 ^x^	9.76	9.89	10.2	9.33 ^x^				
	14	8.37 ^x^	9.88 ^xy^	10.4 ^y^	11.	11.0	9.88	10.1 ^xy^				
	21	11.7 ^y^	10.9 ^y^	11.2 ^y^	9.74	9.89	9.41	10.5 ^y^				
PCV (%)	Overall	33.8	34.3	34.5	34.3	31.6	34.6		1.82	0.840	<0.001	0.239
	7	33.0	32.5	32.2	29.8	31.6	30.4	31.6 ^x^				
	14	35.0	35.8	36.5	38.2	29.3	37.6	35.4 ^y^				
	21	33.3	34.7	34.8	35.1	33.9	35.8	34.6 ^y^				
TEC (×10^6^/mm^3^)	Overall	6.49	6.47	6.44	6.43	6.44	6.40		0.023	0.101	<0.001	0.056
	7	6.57	6.50	6.43	6.41	6.42	6.44	6.46 ^y^				
	14	6.40	6.42	6.41	6.41	6.41	6.31	6.39 ^x^				
	21	6.50	6.47	6.50	6.46	6.49	6.45	6.48 ^y^				
MCV (fL)	Overall	112	119	125	129	118	142		11.8	0.494	<0.001	0.191
	7	90.1	104	121	115	121	110 ^x^	110 ^x^				
	14	140	137	147	152	122	189 ^y^	147 ^y^				
	21	106	117	111	121	111	127 ^x^	116 ^x^				
MCH (pg)	Overall	33.8	32.7	34.8	38.3	38.0	39.5		2.80	0.415	0.004	0.018
	7	30.5	23.9 ^x^	27.9 ^x^	37.7	38.1	36.8	32.5 ^x^				
	14	33.5	37.8 ^y^	40.9 ^y^	43.8	43.4	48.2	41.3 ^y^				
	21	37.3	36.5 ^y^	35.7 ^y^	33.5	32.5	33.6	34.9 ^x^				
MCHC (%)	Overall	31.1	27.1	27.8	29.9	48.8	29.0		1.57	0.767	0.696	0.005
	7	34.0	22.7	23.2	33.1	32.0	33.8	29.8				
	14	24.0	27.6	28.3	28.9	27.4	26.8	36.8				
	21	35.3	30.9	32.0	27.8	29.2	26.3	30.2				
TLC (×10^3^/mm^3^)	Overall	21.3	24.9	22.3	23.4	24.6	23.8		1.47	0.399	<0.001	0.186
	7	21.11	27.8	25.4	29.2	30.8	29.9	27.4 ^z^				
	14	20.9	23.4	18.6	17.3	18.9	16.0	19.2 ^x^				
	21	21.9	23.6	23.0	23.6	24.0	25.6	23.6 ^y^				

Hb = Hemoglobin, PCV = Packed cell volume, TEC = Total erythrocyte count, MCV = Mean corpuscular volume, MCH = Mean corpuscular hemoglobin, MCHC = Mean corpuscular hemoglobin content, TLC = Total leukocyte count, NC = negative control chickens, PC = positive control chickens, coccidia-challenged and non-treated, Sal = coccidia-challenged chickens + salinomycin at 60 mg/kg of feed, BA = coccidia-challenged chickens + benzoic acid at 500 mg/kg of feed, OEO = coccidia-challenged + oregano essential oil at 500 mg/kg of feed, B&O = coccidia-challenged + benzoic acid at 500 mg/kg of feed + oregano essential oil at 500 mg/kg of feed. DPI_m_ = Mean of the days post-infection (DPI), SEM = Pooled standard error of mean. ^x,y,z^ Means with different superscript letters within a column differ significantly (*p* < 0.05) among the DPI (Fisher’s protected least significant difference test). ^1^ Although interactions between treatment and DPI were significant (*p* < 0.05), they were not significant (*p* > 0.05) among the treatment within a DPI.

**Table 5:** Differential leukocyte profiles of broiler chickens supplemented with different anticoccidial agents and challenged with coccidia.

Variables	DPI	Treatment (Trt)						DPI_m_	SEM	*p*-Value		
		NC	PC	Sal	BA	OEO	B&O			Trt	DPI	Trt × DPI
Heterophil (%)	Overall	37.0	39.2	34.4	37.3	35.1	34.9		1.474	0.217	0.242	0.902
	7	37.7	38.3	33.3	35.0	33.0	34.2	35.2				
	14	37.0	40.5	33.0	36.8	35.2	35.0	36.3				
	21	36.3	38.8	37.0	40.3	37.0	35.5	37.5				
Eosinophil (%)	Overall	3.61	2.75	3.42	3.08	2.67	3.58		0.532	0.652	0.295	0.607
	7	2.67	1.75	2.50	3.00	3.25	3.25	2.74				
	14	4.00	3.25	4.50	3.75	2.00	3.00	3.42				
	21	4.17	3.25	3.25	2.50	2.75	4.50	3.40				
Basophil (%)	Overall	0.01	0.08	0.01	0.01	0.17	0.01		0.050	0.120	0.309	0.758
	7	0.01	0.25	0.00	0.01	0.25	0.01	0.08				
	14	0.00	0.00	0.01	0.01	0.25	0.01	0.04				
	21	0.01	0.01	0.01	0.01	0.01	0.01	0.01				
Lymphocyte (%)	Overall	53.2	51.2	54.9	53.3	55.6	54.9		1.544	0.379	0.545	0.976
	7	52.5	52.7	55.3	53.3	54.8	55.3	54.0				
	14	52.7	47.5	54.8	53.0	56.0	54.3	53.0				
	21	54.5	53.2	54.8	53.5	56.0	55.3	54.5				
Monocyte (%)	Overall	6.17	6.83	7.25	6.33	6.58	6.58		0.607	0.808	<0.001	0.685
	7	7.17	7.00 ^xy^	9.00 ^y^	8.75 ^y^	8.75 ^y^	7.25	7.99 ^y^				
	14	6.33	8.75 ^y^	7.75 ^xy^	6.50 ^xy^	6.50 ^xy^	7.75	7.26 ^y^				
	21	5.00	4.75 ^x^	5.00 ^x^	3.75 ^x^	4.50 ^x^	4.75	4.63 ^x^				
Heterophil to Lymphocyte ratio	Overall	0.71	0.78	0.63	0.72	0.64	0.64		0.049	0.268	0.707	0.954
	7	0.73	0.73	0.61	0.68	0.61	0.63	0.67				
	14	0.73	0.86	0.61	0.70	0.64	0.64	0.70				
	21	0.68	0.74	0.69	0.76	0.67	0.65	0.70				

NC = negative control chickens, PC = positive control chickens, coccidia-challenged and non-treated, Sal = coccidia-challenged chickens + Salinomycin at 60 mg/kg of feed, BA = coccidia-challenged chickens + benzoic acid at 500 mg/kg of feed, OEO = coccidia-challenged+ oregano essential oil at 500 mg/kg of feed, B&O = coccidia-challenged + benzoic acid at 500 mg/kg of feed + oregano essential oil at 500 mg/kg of feed DPI_m_ = Mean of the DPI, SEM = Pooled standard error of mean. ^x,y^ Means with different superscript letters within a column differ significantly (*p* < 0.05) among the DPI (Fisher’s protected least significant difference test).

**Table 6:** Enzyme and endocrine profiles of broiler chickens supplemented with different anticoccidial agents and challenged with coccidia.

Variables	DPI	Treatment (Trt)						DPI_m_	SEM	*p*-Value		
		NC	PC	Sal	BA	OEO	B&O			Trt	DPI	Trt × DPI
AST (IU/L)	Overall	112	151	132	125	118	103		18.7	0.565	0.670	0.135
	7	93.6	67.2	169	102	177	94.6	117				
	14	66.8	97.0	89.6	124	84.6	119	118				
	21	176	162	137	149	92.1	94.6	135				
ALT (IU/L)	Overall	18.4	15.7	16.8	19.6	21.4	18.0		4.58	0.962	0.523	0.584
	7	17.6	15.7	18.8	15.7	13.3	18.2	16.6				
	14	17.6	14.5	17.0	21.2	18.8	16.4	20.5				
	21	20.0	17.0	14.5	21.8	14.5	19.4	17.				
ALP (IU/L)	Overall	131	119	142	141	126	132		13.8	0.842	<0.001	0.583
	7	236	179	196	189	203	186	198 ^z^				
	14	98.5	110	127	141	113	127	120 ^y^				
	21	57.7	68.7	103	92.8	61.9	82.5	77.8 ^x^				
T_3_ (ng/mL)	Overall	1.41	1.41	1.38	1.39	1.42	1.40		0.013	0.410	<0.001	0.035
	7	1.36 _b_^x^	1.39 _b_^x^	1.28 _a_^x^	1.36 _b_^x^	1.35 _b_^x^	1.35 _b_^x^	1.35 ^x^				
	14	1.41 ^xy^	1.37 ^x^	1.41 ^y^	1.39 ^xy^	1.38 ^x^	1.35 ^x^	1.38 ^y^				
	21	1.46 ^y^	1.48 ^y^	1.45 ^y^	1.43 ^y^	1.52 ^y^	1.49 ^y^	1.47 ^z^				
T_4_ (µg/dL)	Overall	3.02	3.14	3.01	3.12	2.96	3.18		0.124	0.768	0.221	0.207
	7	3.30 ^y^	3.05	3.10	3.17	2.88	3.23	3.12				
	14	2.82 ^x^	3.23	2.83	3.16	2.84	3.16	3.01				
	21	2.93 ^xy^	3.15	3.11	3.02	3.16	3.15	3.09				
Cortisol (ng/mL)	Overall	1.32	1.49	1.28	1.13	1.19	1.04		0.114	0.131	0.011	0.792
	7	1.31	1.66	1.43	1.35	1.40	1.36	1.42 ^y^				
	14	1.27	1.37	1.38	1.03	1.15	0.94	1.19 ^x^				
	21	1.40	1.44	1.02	1.01	1.00	0.81	1.11 ^x^				

AST = Aspartate Aminotransferase, ALT = Alanine Aminotransferase, ALP = Alkaline Phosphatase, T_3_ = Triiodothyronine, T_4_ = Thyroxine, NC = negative control chickens, PC = positive control chickens, coccidia-challenged and non-treated, Sal = coccidia-challenged chickens + salinomycin at 60 mg/kg of feed, BA = coccidia-challenged chickens + benzoic acid at 500 mg/kg of feed, OEO = coccidia-challenged + oregano essential oil at 500 mg/kg of feed, B&O = coccidia-challenged + benzoic acid at 500 mg/kg of feed + oregano essential oil at 500 mg/kg of feed. DPI_m_ = Mean of the days post-infection (DPI), SEM = Pooled standard error of mean. ^a,b^ Means with different subscript letters within a row differ significantly (*p* < 0.05) among the treatments. ^x,y,z^ Means with different superscript letters within a column differ significantly (*p* < 0.05) among the DPI (Fisher’s protected least significant difference test).

**Table 7:** Biochemical profiles of broiler chickens supplemented with different anticoccidial agents and challenged with coccidia.

Variables (Unit)	DPI	Treatment (Trt)						DPI_m_	SEM	*p*-Value		
		NC	PC	Sal	BA	OEO	B&O			Trt	DPI	Trt × DPI
Glucose (mg/dL)	Overall	122	133	146	133	133	139		3.35	0.082	0.022	0.373
	7	128	134	134	136	140	127 ^x^	133 ^y^				
	14	115	130	129	123	122	130 ^x^	125 ^x^				
	21	123	135	140	140	135	160 ^y^	1397 ^y^				
Total protein (g/dL)	Overall	3.26	3.08	3.08	3.17	3.21	2.92		0.204	0.871	0.010	0.182
	7	2.83	2.92	2.89	2.92 ^x^	2.93	2.29	2.96 ^x^				
	14	3.58	3.06	3.18	3.74 ^y^	3.63	3.01	3.37 ^y^				
	21	3.37	3.26	3.16	2.86 ^x^	3.05	2.47	3.03 ^x^				
Albumin (g/dL)	Overall	1.46	1.35	1.37	1.43	1.44	1.31		0.112	0.896	0.011	0.200
	7	1.26	1.26	1.28	1.28 ^x^	1.29	1.48	1.31 ^x^				
	14	1.64	1.33	1.43	1.70 ^y^	1.63	1.34	1.51 ^y^				
	21	1.49	1.45	1.42	1.30 ^x^	1.40	1.10	1.36 ^x^				
Globulin (g/dL)	Overall	1.79	1.73	1.70	1.75	1.76	1.62		0.115	0.912	0.020	0.262
	7	1.56	1.66	1.61	1.64 ^x^	1.64	1.81	1.65 ^x^				
	14	1.94	1.72	1.75	2.04 ^y^	2.01	1.67	1.86 ^y^				
	21	1.87	1.81	1.74	1.56 ^x^	1.65	1.38	1.67 ^x^				
Albumin-to-globulin ratio	Overall	0.83	0.79	0.81	0.81	0.82	0.82		0.036	0.977	0.941	0.999
	7	0.83	0.77	0.80	0.80	0.80	0.84	0.81				
	14	0.84	0.78	0.82	0.82	0.81	0.81	0.81				
	21	0.81	0.81	0.81	0.81	0.85	0.81	0.81				
Cholesterol (mg/dL)	Overall	87.3	84.5	94.8	86.6	88.4	87.0		3.694	0.495	0.024	0.440
	7	80.0	86.6	104.7 ^y^	89.2	83.8	84.3	88.10 ^xy^				
	14	93.0	85.2	96.6 ^xy^	93.7	97.2	91.9	92.9 ^y^				
	21	88.9	81.7	83.2 ^x^	76.8	84.3	84.9	83.3 ^x^				
Creatinine (mg/dL)	Overall	1.10	0.85	1.05	1.18	1.33	1.07		0.116	0.137	0.242	0.074
	7	1.07	1.10	1.15	1.35	1.25	1.10	1.17				
	14	1.31	0.55	1.20	1.00	1.30	1.00	1.06				
	21	0.92	0.90	0.80	1.20	1.45	1.10	1.06				

NC = negative control chickens, PC = positive control chickens, coccidia-challenged and non-treated, Sal = coccidia-challenged chickens + Salinomycin at 60 mg/kg of feed, BA = coccidia-challenged chickens + benzoic acid at 500 mg/kg of feed, OEO = coccidia-challenged+ oregano essential oil at 500 mg/kg of feed, B&O = coccidia-challenged + benzoic acid at 500 mg/kg of feed + oregano essential oil at 500 mg/kg of feed, DPI_m_ = Mean of the DPI, SEM = Pooled standard error of mean. ^x,y^ Means with different superscript letters within a column differ significantly (*p* < 0.05) among the DPI (Fisher’s protected least significant difference test).

**Table 8:** Fecal oocyst and microbial count, phagocytic activity, and lymphocyte proliferation response of broiler chickens supplemented with different anticoccidial agents and challenged with coccidia.

Variables (Unit)	DPI	Treatment (Trt)						DPI_m_	SEM	*p*-Value		
		NC	PC	Sal	BA	OEO	B&O			Trt	DPI	Trt × DPI
Oocyst count (×10^3^ per gram of feces)	Overall	*	2.70 _b_	1.63 _a_	1.56 _a_	1.51 _a_	1.45 _a_		0.042	0.004	<0.001	0.627
	7	*	2.88	1.67	1.68	1.58	1.56	1.86 ^y^				
	14	*	2.75	1.80	1.73	1.67	1.60	1.90 ^y^				
	21	*	2.47	1.42	1.27	1.25	1.20	1.53 ^x^				
*Escherichia coli* (×10^4^ cfu/mL)	Overall	6.11 _d_	6.64 _e_	5.98 _d_	5.15 _c_	4.91 _b_	3.82 _a_		0.070	<0.001	<0.001	<0.001
	7	5.28 _d_^x^	6.28 _e_^x^	5.00 _d_^x^	3.93 _c_^x^	3.87 _b_^x^	2.77 _a_^x^	4.51 ^x^				
	14	6.09 _d_^y^	6.60 _e_^y^	6.27 _d_^y^	5.20 _c_^y^	4.75 _b_^y^	3.81 _a_^y^	5.45 ^y^				
	21	7.00 _e_^z^	7.03 _e_^z^	6.68 _d_^z^	6.34 _c_^z^	6.11 _b_^z^	4.87 _a_^z^	6.34 ^z^				
*Salmonella* spp. (×10^4^ cfu/mL)	Overall	3.51 _c_	4.70 _d_	3.73 _c_	3.06 _b_	2.93 _b_	2.44 _a_		0.153	<0.001	<0.001	<0.001
	7	4.67 _c_^z^	6.25 _d_^z^	4.46 _bc_^y^	3.41 _a_^y^	3.82 _ab_^y^	4.70 _bc_^z^	4.55 ^z^				
	14	3.34 _c_^y^	4.49 _d_^y^	4.35 _d_^y^	3.61 _c_^y^	2.68 _b_^x^	1.79 _a_^y^	3.38 ^y^				
	21	2.54 _b_^x^	3.35 _c_^x^	2.37 _b_^x^	2.15 _b_^x^	2.28 _b_^x^	0.83 _a_^x^	2.25 ^x^				
*Lactobacillus* spp. (×10^4^ cfu/mL)	Overall	3.84 _b_	3.37 _a_	4.19 _c_	4.94 _d_	5.91 _e_	5.86 _e_		0.081	<0.001	0.107	<0.001
	7	4.04 _b_^y^	3.29 _a_^y^	4.20 _bc_	4.60 _c_^x^	5.83 _d_	5.93 _d_	4.65				
	14	3.78 _b_^x^	2.94 _a_^x^	4.19 _c_	5.00 _d_^y^	5.96 _e_	5.95 _e_	4.64				
	21	3.70 _a_^x^	3.89 _ab_^z^	4.17 _b_	5.22 _c_^y^	5.92 _d_	5.69 _d_	4.76				
Phagocytic activity	Overall	0.87	0.94	0.81	0.72	0.81	0.77		0.069	0.287	<0.001	0.695
	7	0.22	0.21	0.20	0.20	0.19	0.18	0.20 ^x^				
	14	0.97	1.12	0.80	0.87	0.85	0.87	0.91 ^y^				
	21	1.43	1.50	1.42	1.00	1.39	1.28	1.35 ^z^				
Lymphocyte proliferation response	Overall	0.68 _a_	1.08 _b_	0.85 _ab_	0.75 _ab_	0.95 _ab_	1.52 _c_		0.123	0.001	0.065	<0.001
	7	0.56 _a_	1.44 _b_	0.61 _a_	1.05 _ab_	1.04 _ab_	1.01 _ab_	0.95				
	14	0.83	1.24	0.83	0.70	0.60	0.83	0.84				
	21	0.66 _ab_	0.58 _ab_	1.12 _bc_	0.50 _a_	1.22 _c_	1.72 _d_	1.13				

NC = negative control chickens, PC = positive control chickens, coccidia-challenged and non-treated, Sal = coccidia-challenged chickens + salinomycin at 60 mg/kg of feed, BA = coccidia-challenged chickens + benzoic acid at 500 mg/kg of feed, OEO = coccidia-challenged + oregano essential oil at 500 mg/kg of feed, B&O = coccidia-challenged + benzoic acid at 500 mg/kg of feed + oregano essential oil at 500 mg/kg of feed. DPI_m_ = Mean of the days post-infection (DPI), SEM = Pooled standard error of mean, cfu = colony forming unit. ^a,b,c,d,e^ Means with different subscript letters within a row differ significantly (*p* < 0.05) among the treatments. ^x,y,z^ Means with different superscript letters within a column differ significantly (*p* < 0.05) among the DPI (Fisher’s protected least significant difference test). * All values were ‘0’ in NC group; hence, these values were not included in the analysis.

## Data Availability

Data are contained within the article.

## Supplementary Materials

The following supporting information can be downloaded at: https://www.mdpi.com/article/10.3390/ani14203008/s1, Table S1: Sequences of primers used for amplification of target and reference genes. For each gene, the primer sequences for forward (F) and reverse (R) are listed (5′–3′), Melting Point (temperature in °C), the amplicon (product) size (bp), and the NCBI Accession number (Acc) used for the primer design [38].
