# Supplementary material for: Effect of Dietary Benzoic Acid and Oregano Essential Oil as a Substitute for an Anti-Coccidial Agent on Growth Performance and Physiological and Immunological Responses in Broiler Chickens Challenged with Eimeria Species
Source: Animals (Basel). 2024 Oct 17;14(20):3008. doi: 10.3390/ani14203008 (PMC11504159; doi:10.3390/ani14203008)
Supplement: Supplementary file 1 [file animals-14-03008-s001.zip › animals-3226138-supplementary.pdf]

Table S1. Sequences of primers used for amplification of target and reference genes. For each gene, the primer sequences for forward (F) and reverse (R) are listed (5'-3'), Melting Point (temperature in °C), the amplicon (product) size (bp) and the NCBI Accession number (Acc) used for the primer design.

Gene	F/ R	Primer Sequence	Melting Point (°C)	Product Size (bp)	Accession (reference)	References
IL-10	F	CGCTGTCACCGCTTCTTCA	58.0	63	NM_001004414.2	Calik *et al*. (2022)
	R	CGTCTCCTTGATCTGCTTGATG	55.6			
IFN-γ	F	AGTCAAAGCCGCACATCAAAC	56.4	63 82	NM_205149.1	Calik *et al*. (2022)
	R	TTCACCTTCTTCACGCCATCA	56.7			
TLR-4	F	CCACACACCTGCCTACATGAA	57.3	63	NM_001030693	Calik *et al*. (2022)
	R	GGATGGCAAGAGGACATATCAAA	54.9			
GAPDH	F	CCTAGGATACACAGAGGACCAGGTT	59.2	64	NM_204305	Calik *et al*. (2022)
	R	GGTGGAGGAATGGCTGTCA	57.3			

IL: Interleukin; IFN-γ: Interferon gamma; TLR: Toll Like Receptor; GAPDH: Glyceraldehyde 3-phosphate dehydrogenase.

Calik, A.; Nima. K.E.; Schyns, G.; White, M.B.; Walsh, M.C.; Romero, L.F.; Dalloul, R.A. Influence of dietary vitamin E and selenium supplementation on broilers subjected to heat stress, Part II: oxidative stress, immune response, gut integrity, and intestinal microbiota. *Poult. Sci.* **2022**,*101,* 101858. https://doi.org/10.1016/j.psj.2022.101858
